# The association of adherence to 24-hour movement guidelines with frailty and mortality: cross-sectional and longitudinal analyses of NHANES data

**DOI:** 10.1186/s44167-024-00056-6

**Published:** 2024-07-05

**Authors:** Daniel J. Meister, D. Scott Kehler, Danielle R. Bouchard, Amy M. Thomson, Martin Sénéchal

**Affiliations:** 1 Cardiometabolic Exercise & Lifestyle Laboratory, University of New Brunswick, 90 MacKay Dr Room: 105, Fredericton, NB E3B 5A3 Canada; 2 Faculty of Kinesiology, University of New Brunswick, 90 MacKay Dr Room: 105, Fredericton, NB E3B 5A3 Canada; 3 School of Physiotherapy, Dalhousie University, 5869 University Ave, Halifax, NS B3H 4R2 Canada; 4 Division of Geriatric Medicine, Dalhousie University, 5869 University Ave, Halifax, NS B3H 4R2 Canada

**Keywords:** Aging, Healthy aging, Movement Behavior, 24 Hour Movement guidelines, Frailty

## Abstract

**Background:**

Adherence to the Canadian 24-Hour Movement Guidelines (24 H-MG) has been associated with a reduced risk of developing various chronic conditions. However, its association with frailty and all-cause mortality has not been investigated. Therefore, our primary and secondary objective was to investigate the association between adherence to the 24 H-MG and frailty and mortality, respectively.

**Methods:**

This study included 2739 individuals (age = 50.6 ± 18.1 years; male = 1370 (50.0%)) from the 2005–2006 cycle of the National Health and Nutrition Examination Survey (NHANES). Frailty was quantified with a 46-item frailty index and analyzed cross-sectionally using linear regression. All-cause mortality data were obtained from the National Death Index and was analyzed prospectively over 10 years using Cox regression. The primary exposure variable was six individual and combined 24 H-MG components including the moderated-to-vigorous physical activity, light physical activity, sedentary time, recreational screen time, sleep, and strength training guidelines. All analyses were stratified into two age groups (younger: 20–64 and older adults 65 + years).

**Results:**

Our cross-sectional analyses demonstrated an inverse dose-response relationship between the number of individual 24 H-MG components met and frailty level in adults aged 20–64 (β = -0.439 (95% C.I. = -0.551:-0.328)) and 65+ (β = -0.322 (95% C.I. = -0.490:-0.154)). Of the individual guideline components, following the moderate-to-vigorous physical activity (MVPA) guideline in individuals aged 20–64 and the recreational screen time guideline in adults aged 65 + was associated with lower frailty (*p* < 0.001). There was no clear prospective relationship between adherence to the combined 24 H-MG and mortality. Of the individual guideline components, only meeting the MVPA guideline component in the 65 + group was prospectively associated with reduced mortality risk (HR = 0.48 (95% C.I. = 0.25–0.93)).

**Conclusion:**

Adherence to the Canadian 24 H-MG may be protective against frailty. Increasing MVPA and decreasing recreational screen time may be important behaviors to consider for frailty prevention and should be investigated further.

## Introduction

In 2020, the 24-Hour Movement Guidelines (24 H-MG) for adults aged 20–65 and adults aged 65 years or older [1] were introduced to the public to complement previously released guidelines for children and youth (aged 5–17) [2], and the early years (aged 0–4) [3]. These guidelines recommend ≥ 150 min of moderate-to-vigorous physical activity (MVPA) per week; muscle-strengthening activities at least twice per week; several hours of light physical activity (LPA) per day (including standing); 7–9 h of sleep per night for adults aged 20–64 and 7–8 h of sleep per night for adults aged 65+; ≤ 3 h of recreational screen time per day; and ≤ 8 h of sedentary time per day.

These new guidelines were implemented based on the evidence that other types of behaviors beyond MVPA, including sleep [4], and sedentary behavior [5], are associated with morbidity and mortality in adults. Therefore, considering movement behavior over a full 24-hour period [6] appears to be relevant when promoting health, as opposed to focusing exclusively on MVPA, which generally represents < 5% of the total day [7]. Studies have investigated the association between 24-hour movement behavior and several outcomes, such as obesity [8], global cognition in children [9], and mortality risk [6, 10]. However, to the best of our knowledge, no research has been conducted to investigate the relationship between adherence to the 24 H-MG and frailty, or whether adherence to the 24 H-MG influences mortality when accounting for frailty.

Frailty is an age-related health state of reduced physiologic reserve across multiple body systems which leads to reduced capacity to deal with stressors [11–13]. Thus, individuals living with frailty are at an increased risk of adverse health outcomes [14–17]. Although the complete 24 H-MG have not been studied in relation to frailty, studies suggest that its individual components are associated with frailty. For example, even individuals who meet less than 50% of the weekly MVPA recommendation have been shown to have lower frailty compared to individuals who do not do any MVPA [18]. Sleeping less than 6 h a night or sleeping more than 8 h a night is associated with a 13% and 21% increase in risk of frailty, respectively [19]. In addition, increased sedentary behavior has been shown to increase one’s risk of being frail [18, 20], and evidence suggests that replacing an equivalent sedentary time with 113 min of LPA or 41 min of MVPA per day can reduce frailty by a clinically significant amount of at least one deficit point [21]. Sedentary time [22], physical activity levels [23], and sleeping time [24] have also all been shown to influence mortality independent of frailty levels. However, there is no study yet that has investigated the association between the comprehensive 24 H-MG and frailty or the 24 H-MG and mortality when controlling for frailty.

Consequently, the first objective of this study was to investigate the cross-sectional association between adherence to the new Canadian 24 H-MG, its individual components and frailty in adults and older adults. It was hypothesized that adherence to the combined 24 H-MG, and the individual components of the 24 H-MG, will be associated with lower frailty levels. The secondary objective was to investigate the prospective association between adherence to the complete Canadian 24 H-MG, its individual components, and mortality when accounting for frailty. It was hypothesized that adherence to the complete Canadian 24 H-MG and adherence to its individual components will be associated with reduced mortality regardless of frailty.

## Methods

### Study design

Data from the 2005–2006 National Health and Nutrition Examination Survey (NHANES) cycle was used [25]. This cycle was chosen because it was the only publicly available cycle that contained physical activity (PA) measured using accelerometry and self-reported screen time, strength training, and sleep data at the time of this analysis. NHANES is a cross-sectional survey with participants selected via stratified random sampling across the United States. However, by using the National Death Index (NDI), we were able to prospectively examine whether meeting the 24 H-MG at baseline was associated with all-cause mortality risk. Although frailty prevalence increases with age, frailty has been observed in younger adults [26]. Thus, our analysis included adults above 20 years of age to investigate the relationship between adherence to the 24 H-MG and frailty across the adult lifespan. Participants were excluded from the analysis if they were missing valid accelerometry data, enough data to construct a frailty index (FI) or covariate data. As defined in previous studies [27], accelerometry data were considered valid if the participant had 4 days of at least 10 h of wear time.

**Fig. 1:**
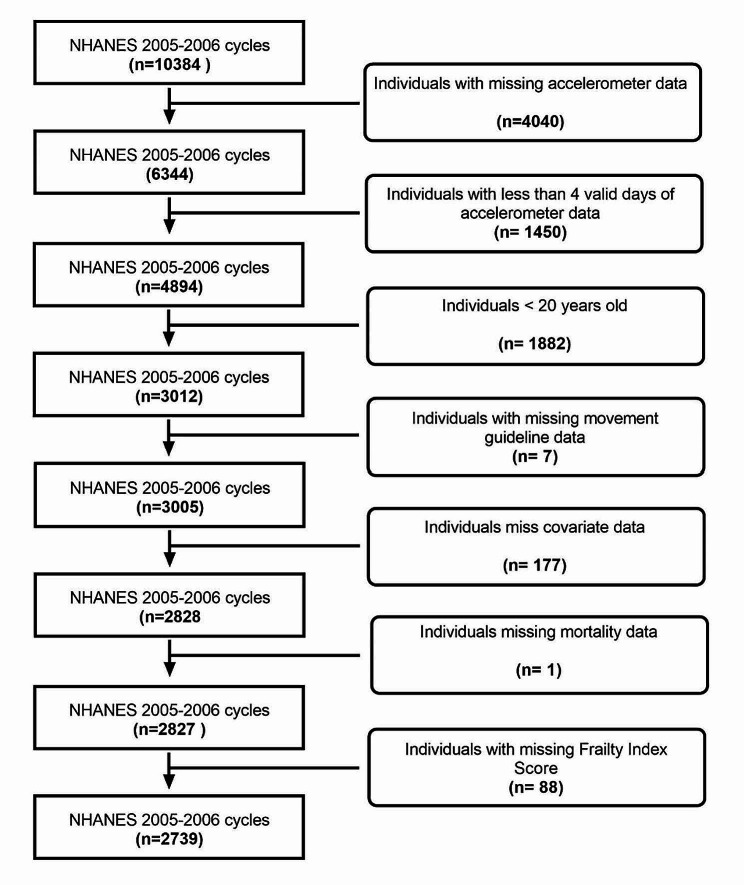
Final sample flow chart

Originally, the 2005–2006 NHANES database contained 10,384 records. Of those, 4040 were missing accelerometer data, and 1450 did not have valid accelerometer data. Of the 4894 remaining individuals an additional 1882 did not meet the age requirements (≥ 20) leaving 3012 remaining individuals. An additional 7 individuals were missing 24 H-MG data (sleep and recreational screen time use), 177 were missing data related to confounding variables (alcohol use and education level), 1 was missing mortality data, and 88 were missing the necessary data to construct a FI. The final sample size of the current study was 2739 individuals (Fig. 1). NHANES was approved by the National Center of Health Statistics Institutional Review Board and each participant provided written consent.

### Primary exposure variables

#### Physical activity and sedentary time

PA and sedentary time were quantified using accelerometer data. Participants wore an accelerometer on their right hip. Uniaxial ActiGraph AM-7164 accelerometers were used, and intensity readings were summed over each 1-minute epoch in counts per minute. Participants were instructed to wear the accelerometer during waking hours for seven consecutive days and to remove it at night and during water-based activities (e.g., bathing, and swimming). The data were considered valid if the accelerometer had been worn for a minimum of four days independent of week or weekend days, with a minimum average wear time of 10 h per day [27]. Non-wear time was identified by at least 60 consecutive minutes of counts between 0 and 100 [27]. Sedentary time, MVPA, and LPA, were identified using age and sex specific count per minute cut-points previously used by Troiano, et al. [27].

Adherence to three of the guideline components were quantified using these data. First, the MVPA guideline component recommends ≥ 150 min of MVPA per week [28]. Second, the 24 H-MG further stipulate that individuals should perform several hours of structured and unstructured LPA, with the premise that more is better, but do not provide a specific recommendation for the number of hours. Therefore, time in LPA was quantified, and quartiles for the study sample were computed stratified by age categories (20–39, 40–59, 60+) to ensure an appropriate distribution of participants of varying ages across the quartiles [8]. The highest quartile in each age group were then combined and used as the criterion for reaching this guideline. Third, the sedentary time guideline component recommends ≤ 8 h per day spent sedentary [1].

#### Resistance training

Two questions were asked in NHANES regarding resistance training. First, participants were asked “Over the past 30 days, did {you/Sample Person (SP)} do any physical activities specifically designed to strengthen {your/his/her} muscles such as lifting weights, push-ups or sit-ups? Include all such activities even if you have mentioned them before.” If they responded yes to this question then respondents were prompted with the following question: “[Over the past 30 days], how often did {you/SP} do these physical activities? [Activities designed to strengthen {your/his/her} muscles such as lifting weights, push-ups or sit-ups]”. The answers to this second questions were divided by 30 and multiplied by seven to quantify number of bouts of resistance training performed per week, while those who answered “no” to the first question were assumed to not perform any resistance training. The 24 H-MG recommend that individuals should perform ≥ 2 sessions of resistance training per week [1].

#### Sleep

Sleep was self-reported using the following question “The next set of questions is about your sleeping habits. How much sleep {do you/does SP} usually get at night on weekdays or workdays?”, with answers ranging from 1 to 11 h/night. Participants sleeping the recommended hours within their age category of the 24 H-MG (7 to 9 h for adults aged 20–64, and 7 to 8 h for adults aged 65 [1]) met the sleep guideline, whereas participants sleeping either more or less hours per night did not.

#### Recreational screen time

Recreational screen time was also self-reported. Participants were asked the question “Over the past 30 days, on average about how many hours per day did {you/SP} sit and watch TV or videos? Would you say…” with answers being Less than 1 h, 1 h, 2 h, 3 h, 4 h, or 5 h or more. NHANES participants were also asked how much time they spent using the computer. We only used data from the TV viewing question because it was impossible to separate recreational and productive screen time in the computer usage question. For recreational screen time, participants were found to meet this guideline component if they reported watching ≤ 3 h of TV per day.

#### The 24 H-MG

The new 24 H-MG for adults 20–64 and 65 + from the Canadian Society for Exercise Physiology [1] were adapted to create a comprehensive movement behavior assessment. The guidelines include recommendations for PA, sitting time, and sleep. Based on the guidelines, a single dichotomous variable was created for each of the 6 components (MVPA, LPA, sedentary time, recreational screen time, sleep, and resistance training). For each guideline component, a 1 was given to the individuals when meeting the guideline for this criterion and 0 was given for not meeting the guideline. To investigate the association between the combined 24 H-MG and frailty, everyone’s dichotomous variable of individual guideline was combined into a composite score: the total 24 H-MG. Scores ranged from 0 to 6; zero represents not meeting any of the guideline components and six represents meeting the complete 24 H-MG. For all regression models, those meeting five or six of the guidelines were combined into one group due to the small number of individuals who met all the guidelines (*n* = 16).

#### Co-variates

Co-variates considered included age, sex, ethnicity, education level, and smoking and alcohol usage, all of which have been shown to influence frailty [29–31]. All co-variates were self-reported. Ethnicity was categorized as either Non-Hispanic White, Non-Hispanic Black, Mexican American, or other. Education level was categorized as either less than grade 11; high school diploma or some college or AA degree; or college graduate or above. For smoking status, participants were categorized as non-smokers if they had smoked less than 100 cigarettes in their lifetime, past smokers if they had smoked more than 100 cigarettes in lifetime but had since quit, and current smokers if they had smoked more than 100 cigarettes in lifetime and were still smoking, as per the NHANES classifications [32]. For alcohol consumption, individuals were classified as either non/light drinkers (less than 1 drinks/day), moderate drinkers (1–2 drinks/day) and heavy drinkers (> 2 drinks/day).

### Primary outcome

#### Frailty

Frailty levels were defined using The Accumulation of Health Deficits Model [33]. This model has been operationalized to create a FI which quantifies frailty by dividing the number of deficits observed by the total number of potential deficits measured [33]. In the current study, we used the 46-item deficit model developed and validated for NHANES [34], which included a combination of self-reported and objectively measured data spread through 5 main categories: comorbidities, functions, signs and symptoms, laboratory values, and others self-reported information [34]. Individuals who had a minimum of 80% of the 46-item were included in the analysis. As an example, if someone had 19 of the 46 items measured being identified as deficits, their FI score would be 19/46 or 0.442.

#### Mortality

Mortality-Death Certificate records from the National Death Index (NDI) were linked with records from the NHANES database. These records provide information regarding the cause of death, as well as the time to death recorded in months from the day the participant’s examination occurred with a total follow up time of 10 years. For this study, we focused on all-cause mortality. Months to death was converted to years to death by dividing months to death by 12. The data were obtained from the NHANES website where additional documentation regarding the dataset can be found [35].

### Statistical analysis

Descriptive data were reported using mean ± standard deviation (SD) for continuous variables and n (%) for categorical variables. An independent samples t-test and chi-squared test were used to compare the age and sex differences, respectively, between the included and excluded samples and two-sample t-tests and chi square tests were used to investigate the difference between age groups for continuous and categorical variables respectively.

For the linear regression, a box-cox transformation was applied to the data so that it met the normality assumption. Multiple linear regression was used to investigate the association between adherence to the 24 H-MG and frailty, and to investigate the association between adherence to each of the individual guideline components and frailty. Linear regression results were reported as β-values with standard error. Cox proportional hazard models were then created to estimate the risk of all-causes mortality based on the composite score of the complete 24 H-MG, as well as meeting the individual guideline components. Cox proportional hazard results were reported as hazard ratios with 95% confidence intervals and used a group of individuals that did not meet any of the 24 H-MG as a reference. All models were adjusted for age, sex, ethnicity, education level, cigarette, and alcohol usage. Cox regression model adjusted for all co-variates and frailty was also run. Since an interaction term between age and the 24HMG was found to be significant for age (*p* < 0.05), all regression models were run by age group (20–64 and 65 + yrs). Linear regression models and Cox proportional hazard models were run per individual guidelines, or the number of guideline components met. Data management and statistical analysis were performed using RStudio software version 4.1.2. An alpha level of 0.05 was used for all analyses. Statistical analyses accounted for sample weights.

## Results

Our sample consisted of 2739 individuals with an average age of 50.6 ± 18.1 years, 1370 (50.0%) of whom were male (Table 1). The average FI of our sample was 0.122 ± 0.104, while on average, our sample met 2.63 ± 1.29 guideline components. Participants who were excluded (*n* = 2240) from analyses were significantly younger (45.58 ± 19.9 vs. 50.6 ± 18.1 years) and less proportionately male (45.4% vs. 50%).

**Table 1:** General characteristics of the study sample using the 2005–2006 NHANES cycle

	Total (*n* = 2739)	Adults Aged 20–64 (*n* = 2024)	Adults Aged 65+ (*n* = 715)	
Age (years)	50.6 ± 18.1	42.2 ± 12.7	74.3 ± 6.4	**< 0.001**
Male n (%)	1370 (50.0%)	992 (49.0%)	378 (52.9%)	0.076
**Ethnicity**				
Non-Hispanic white n (%)	1445 (52.7%)	967 (47.8%)	478 (66.9%)	
Non-Hispanic black n (%)	560 (20.4%	436 (21.5%)	124 (17.4%)	
Mexican American n (%)	560 (20.4%)	473 (23.4%)	87 (12.2%)	**< 0.001**
Other	174 (6.3%)	149 (7.4%)	25 (3.5%)	
**Education**				
Grade 11 or Below n (%)	691 (25.2%)	450 (22.2%)	241 (33.8%)	
High School Diploma/Some College/AA Degree n (%)	1460 (53.3%)	1113 (55.0%)	347 (48.6%)	**< 0.001**
College Graduate/Above n (%)	588 (21.47%)	462 (22.8%)	126 (17.6%)	
**Smoking Status**				
Current Smoker n (%)	522 (19.1%)	448 (22.1%)	74 (10.4%)	
Past Smoker n (%)	774 (28.3%)	447 (22.1%)	327 (45.8%)	**< 0.001**
Non-Smoker n (%)	1442 (52.7%)	1130 (55.8%)	313 (43.8%)	
**Alcohol Use**				
Heavy Drinker n (%)	629 (23.0%)	567 (28.0%)	62 (8.7%)	
Moderate Drinker n (%)	503 (18.4%)	412 (20.4%)	91 (12.7%)	**< 0.001**
Light/Non-Drinker n (%)	1607 (58.7%)	1046 (51.7%)	561 (78.6%)	
**Frailty**				
Frailty Index mean (0–1)	0.122 ± 0.104	0.094 ± 0.084	0.202 ± 0.111	**< 0.001**
**Movement Guidelines**				
Guideline components followed	2.63 ± 1.29	2.85 ± 1.25	2.01 ± 1.21	**< 0.001**
**Mortality**				
Death n (%)	391 (14.3%)	119 (5.9%)	272 (38.0%)	**< 0.001**

Note. Data are presented as mean ± SD for continuous variable and n (%) for categorical variables

Table 2 describes how many individuals were meeting each individual guideline components, as well as how many guideline components individuals were following split by age group. The recreational screen time guideline component was the most followed (*n* = 2106 (76.9%)), while the strength training guideline component was the least followed (*n* = 512 (18.7%)). The complete 24 H-MG were met by 16 (0.6%) individuals and 111 (4.1%) individuals did not meet any of the movement guidelines. The most common number of guideline components followed was 3 (*n* = 725 (26.5%)).

**Table 2:** Number of individuals meeting movement guidelines from the 2005–2006 NHANES cycle

	Total	Adults Aged 20–64	Adults Aged 65+	
	Number of People n (%)	Number of People n (%)	Number of People n (%)	
**Individual Guidelines**				
MVPA Guideline	998 (36.4%)	914 (45.1%)	84 (11.8%)	**< 0.001**
Light Physical Activity Guideline	688 (25.1%)	528 (26.1%)	160 (22.4%)	0.055
Sedentary Time Guideline	1286 (47.0%)	1077 (53.2%)	209 (29.3%)	**< 0.001**
Recreational Screen Time Guideline	2106 (76.9%)	1640 (81.0%)	466 (65.3%)	**< 0.001**
Sleep Guideline	1617 (59.0%)	1224 (60.4%)	393 (55.0%)	**0.011**
Strength Training Guideline	512 (18.7%)	392 (19.4%)	120 (16.8%)	0.142
**# of Guidelines Met**				
0 Guidelines	111 (4.1%)	49 (2.4%)	62 (8.7%)	**< 0.001**
1 Guideline	456 (16.7%)	255 (12.6%)	201 (28.2%)	
2 Guidelines	713 (26.0%)	484 (23.9%)	229 (32.1%)	
3 Guidelines	725 (26.5%)	591 (29.2%)	134 (18.8%)	
4 Guidelines	536 (19.6%)	464 (22.9%)	72 (10.1%)	
5 Guidelines	182 (6.6%)	169 (8.4%)	13 (1.8%)	
6 Guidelines	16 (0.6%)	13 (0.6%)	3 (0.4%)	

Note. MVPA = moderate-to-vigorous physical activity

The results of the linear regression analysis (Table 3) showed that meeting at least one of the guideline components in the 20–64 group (β = -0.144 (95% C.I. = -0.252: -0.035); *P* = 0.009) and at least 2 components in the 65 + group was associated with a lower FI score (β = -0.111 (95% C.I. = -0.193:-0.029); *P* = 0.008). Meeting each of the individual guideline components was associated with a lower FI score, except for the sleep guidelines in the 65 + group (β = -0.035 (95% C.I. = -0.083:0.012); *P* = 0.094). Meeting the MVPA guideline component had the strongest association with lower FI in the 20–64 age group (β = -0.144 (95% C.I. = -0.183: -0.106; P = < 0.001), while meeting the recreational screen time guideline component had the strongest association with FI in the 65 + age group (β = -0.131 (95% C.I. = -0.181: -0.081); P = < 0.001).

**Table 3:** Linear regression analysis looking at the relationship between adherence to the Canadian 24-Hour Movement guidelines, it’s individual components and frailty from the 2005–2006 NHANES cycle

		Adults Aged 20–64						Adults Aged 65+			
	β-Value		95% CI		R^2^	*P*-value	β-Value	95%CI		R^2^	*P*-value
**Individual Guidelines**											
MVPA Guideline	-0.144		-0.183	-0.106	0.26	**< 0.001**	-0.093	-0.174	-0.012	0.11	**0.002**
Light Physical Activity Guideline	-0.090		-0.131	-0.050	0.22	**< 0.001**	-0.109	-0.165	-0.053	0.11	**< 0.001**
Sedentary Time Guideline	-0.070		-0.108	-0.032	0.23	**< 0.001**	-0.051	-0.104	0.001	0.10	**0.021**
Recreational Screen Time Guideline	-0.138		-0.191	-0.086	0.25	**< 0.001**	-0.131	-0.181	-0.081	0.14	**< 0.001**
Sleep Guideline	-0.041		-0.078	-0.004	0.22	**0.013**	-0.035	-0.083	0.012	0.11	0.094
Strength Training Guideline	-0.040		-0.087	0.007	0.22	**0.038**	-0.099	-0.157	-0.041	0.10	**< 0.001**
**# of Guideline Components Met**											
1 Guideline Component	-0.144		-0.252	-0.035	0.25	**0.009**	-0.026	-0.105	0.054	0.12	0.534
2 Guideline Components	-0.309		-0.412	-0.206	0.22	**< 0.001**	-0.111	-0.193	-0.029	0.10	**0.008**
3 Guideline Components	-0.344		-0.445	-0.244	0.23	**< 0.001**	-0.162	-0.248	-0.076	0.10	**< 0.001**
4 Guideline Components	-0.373		-0.476	-0.270	0.23	**< 0.001**	-0.242	-0.336	-0.148	0.11	**< 0.001**
5–6 Guideline Components	-0.439		-0.551	-0.328	0.23	**< 0.001**	-0.322	-0.490	-0.154	0.10	**< 0.001**

Note. All models are adjusted for age, sex, education, race, smoking status, and alcohol use. MVPA = moderate-to-vigorous physical activity. One model was run per individual guidelines, or the number of guideline components met. Bolded text represents statistical significance (*P* < 0.05)

**Fig. 2:**
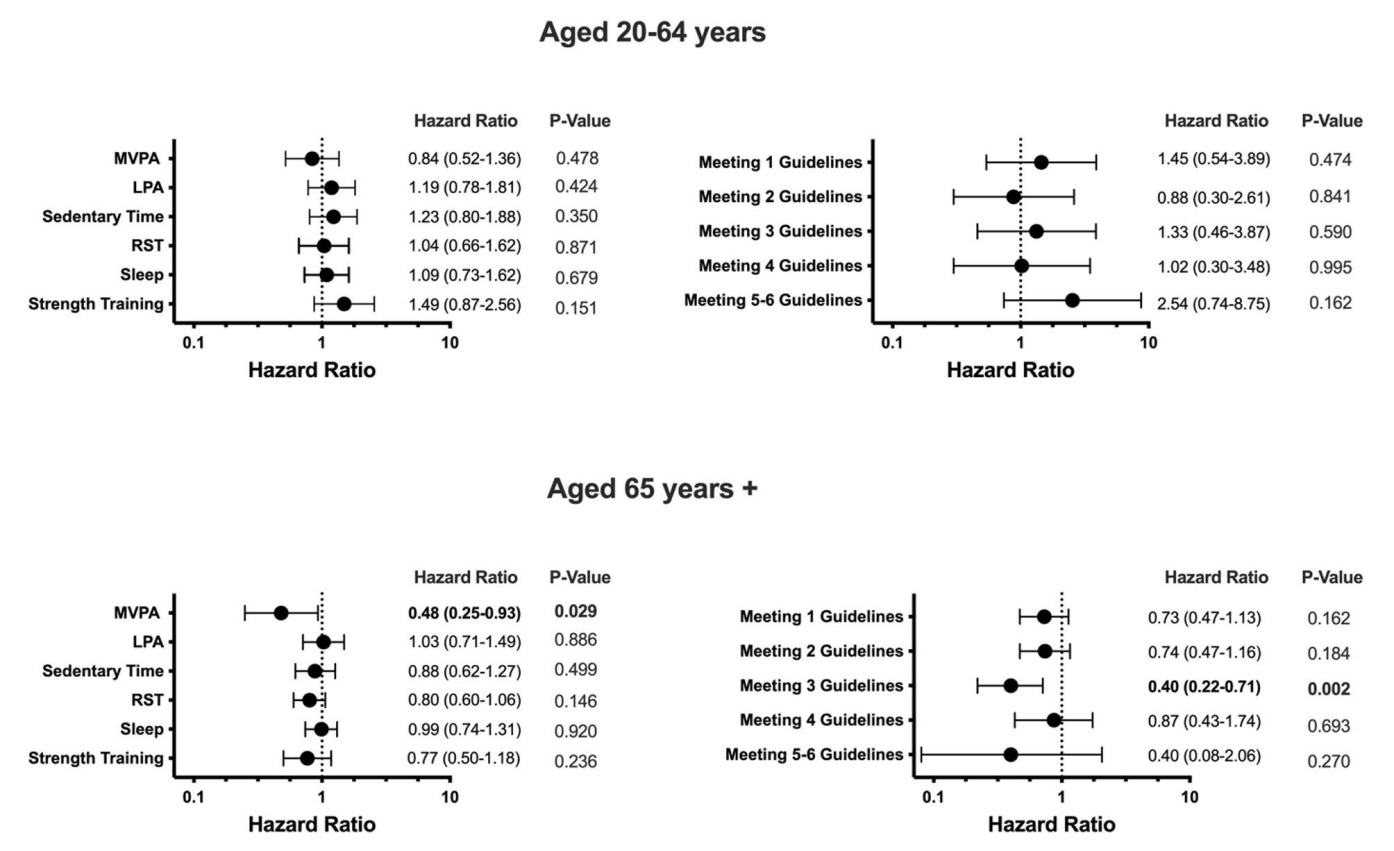
Cox Regression results looking at the relationship between adherence to the Canadian 24-Hour Movement Guidelines, individual guideline components, and mortality. All analyses were adjusted for age, sex, ethnicity, education level, cigarette, alcohol usage, and frailty levels. Bolded text represents statistical significance (*p* < 0.05). MVPA = Moderate to Vigorous Physical Activity; LPA = Light Physical Activity; and RST = Recreation Screen Time

In the 20–64 age group, 119 (5.9%) individuals died. The Cox Regression model showed no association between adherence to the guidelines or the individual guideline components and mortality. This lack of association remained once frailty was added to the model (Fig. 2). Frailty was significantly associated with mortality (for every 0.01 increase HR = 1.05 (95% C.I. = 1.04–1.07) P = < 0.001) in the younger age group.

In the 65 + age group, 272 (38%) individuals died. When adjusting for known co-variates, Cox Regression model showed that only meeting 2 (HR = 0.60 (95% C.I. = 0.38–0.93) *P* = 0.022) or 3 guidelines (HR = 0.32 (95% C.I. = 0.18–0.58) P = < 0.001), and meeting the MVPA (0.45 (95% C.I. = 0.22–0.91) *P* = 0.025) and recreational screen time guideline (HR = 0.67 (95% C.I. = 0.51–0.90) *P* = 0.007) components individually was associated with reduced risk of mortality. Once the model was further adjusted for frailty (Fig. 2), only meeting 3 guideline components (HR = 0.40 (95% C.I. = 0.22–0.71); *P* = 0.002) was associated with reduced mortality. Of the individual guidelines, only meeting the MVPA guideline component (HR = 0.48 (95% CI = 0.25–0.93); *P* = 0.02) was associated with reduced risk of mortality. Frailty was also associated with increased risk of mortality (for every 0.01 increase HR = 1.04 (95% C.I. = 1.03–1.05) P = < 0.001).

## Discussion

The current study provides relevant insights for the management and the treatment of frailty in adults and older adults. First, we found that in adults and older adults meeting at least one or two components of the 24 H-MG was associated with lower frailty. Second, the MVPA and recreational screen time guideline components were the most strongly associated with lower frailty in younger adults compared to recreational screen time and LPA in older adults. Third, only adults aged 65 + years meeting 3 guideline components had a lower risk of premature all-cause mortality, with following the MVPA guideline component being the strongest predictor of all-cause mortality. These results are important because they provide insight into the possibility of implementing the 24 H-MG as a strategy to prevent or treat frailty in adults aged (20–64 years) and older adults (65 + years).

Our linear regression results show that adhering to at least one guideline component in the 20–64 age group and at least two guideline components in the 65 + group was associated with lower FI score and there was a dose response relationship between the number of guideline components met and reduction in FI score. This adds to the growing body of evidence that behavior over the 24-hour day is important to health [6, 8–10]. These results also support past studies that suggest replacing sedentary time or LPA with MVPA helps prevent frailty [36–39] as (1) MVPA remained significantly associated with FI when adjusting for either LPA or sedentary time, and (2) MVPA presented with a stronger beta than sedentary time or LPA (results not shown). Overall, our data are important since they help better understand the relationship between the 24 H-MG and frailty on the whole spectrum of age affected by frailty.

Except for the sleep guideline component in the 65 + age group, adherence to each individual guideline component in both the 20–64 and 65 + age group was also associated with lower FI score. Of the individual guideline components, meeting the MVPA guideline component in the 20–64 age group and the recreational screen time guideline component in the 65 + age group was associated with the lowest FI score making these components the strongest predictors of frailty in each age group, respectively. This supports past studies that found that performing MVPA prevents frailty [21] in both males and females [18, 40] and when considering both bout and sporadic MVPA [18]. Our study adds to these results by showing that performing MVPA is associated with lower FI score in both a younger (20–64) and older (65+) sample, whereas most past studies have only considered older adults (50+).

Our results also agree with previous studies that found that increased sedentary time is associated with greater frailty [20] and that recreational screen time may be more detrimental than other sedentary behavior when considering frailty prevention [41]. As recreation screen time is associated with less MVAP and increased weight, behavioural problems, anxiety, and psychological health problems, there may be an additive effect for that increases this risk of frailty [42–44]. In addition, recreation screen time, in particular TV viewing, has been associated with changes in brain structure, predisposing individuals for cognitive impairments, a potential feature of frailty [42]. These results are relevant because they suggest that clinicians who are using the 24 H-MG to prevent frailty should specifically emphasize the potential harm associated with recreational screen viewing.

Meeting the sleep length guideline component was associated with lower frailty in the 20–64 age group. This confirms results from a systematic review that found that both long (> 8 h) and short (< 6 h) sleep duration was predictive of frailty [19]. Nevertheless, other studies have found no association between sleep duration and frailty [45], which align with our lack of association observed in individuals aged 65 +. In the systematic review cited above [19] they used the cut offs of < 6 h of sleep a night or > 8 h of sleep a night to investigate the relationship between sleep duration and frailty. They found that long sleep duration increased frailty risk by more (21%) than short sleep duration (13%). It is possible that the range of sleep duration included in the 24 H-MG for adults 65+ (7–8 h) is too narrow and sleep durations such as 6 h may still be enough to prevent frailty. In addition, the 24 H-MG contain the recommendation that sleep timing should be consistent. This could not be measured in our study as it was not recorded in NHANES. Nevertheless, sleep timing and different extremes of sleep duration should be considered in future studies to better understand frailty prevention.

In both the 20–64 and 65 + age group in our study, meeting the resistance training guideline component was associated with reduced frailty. International clinical practice guidelines released in 2019 recommend progressive resistance training as part of a frailty management plan [12]. However, to the best of our knowledge, past studies investigating the relationship between combined movement behavior and frailty mostly focus MVPA without including resistance training [21, 40]. Therefore, our research adds to the scientific literature by including resistance training in our analysis and demonstrates that performing resistance training as recommended by the 24 H-MG is associated with lower frailty. To better understand frailty prevention, future studies should investigate the optimal number of sessions of resistance training per week needed to prevent frailty.

A secondary finding of our study is that there was no clear relationship between adherence to the 24 H-MG and risk of all-cause mortality when controlling for frailty. Only meeting three guideline components in the 65 + age group was associated with reduced mortality, while meeting more than three did not protect against mortality. This result was surprising considering the premise of the 24 H-MG suggesting that all behavior across the day matters. However, this finding could be explained by the fact that a very small number of participants met all the guidelines components, which impacted the confidence intervals. For example, only 7.2% of our sample met five or six guidelines’ components, which could explain the results observed. Nevertheless, to the best of our knowledge, this is the first study investigating the relationship between 24 H-MG and mortality while controlling for frailty. Two other studies have looked at the relationship between 24 H-MG and mortality [6, 10]. While both studies found that daily movement behavior composition is related to mortality, neither controlled for frailty, a known predictor of mortality [17]. These studies also did not consider recreational screen time or strength training, which are important predictors of mortality [1].

Of our individual guideline components, only the MVPA guideline component in the 65 + age group was significantly associated with reduced mortality. A past study investigating mortality, frailty and movement behavior found that replacing sedentary time with 38 min–18 min per day of MVPA in vulnerable and mildly frail individuals, respectively, reduced risk of mortality by 50%, but replacing sedentary time with MVPA did not impact mortality in non-frail or moderately-severely frail individuals [21]. Interestingly, our 20–64 age group had an average FI of 0.094 (non-frail) while our 65 + age group had a FI of 0.202 (mildly frail) meaning that the average FI and association between MVPA and risk of mortality are similar between the two studies. However, the study also found that replacing sedentary time with LPA reduced risk of mortality in mildly and moderately-severely frail individuals [21], which was not supported by our study as LPA was not associated with mortality. This difference could be explained by the way that LPA was quantified in the two studies. In our study, we identified whether individuals had met the LPA guideline component based on quantiles, whereas the previous study [21] used isotemporal substitution models to investigate how replacing sedentary time with LPA impacted frailty. Additionally, based on the current literature, our sample of young individuals was quite functional despite presenting frailty deficit features accumulation. Meanwhile, the older age group exhibited serious frailty deficits accumulation and was more advanced on the frailty continuum, although still functional. This distinction is important to consider when interpreting our results as our sample may differ from participants of other studies who might exhibit a more severe frailty level. Once clear recommendations are determined on how much LPA an individual should accumulate, the relationship between meeting this guideline, frailty, and mortality should be revisited.

Several limitations of this study need to be discussed. First, recreational screen time, strength training, and sleep time were self-reported and might have influenced the association observed. Recreational screen time also limited to TV viewing time as recreational and productive computer usage could not be differentiated, potentially impacting the number of individuals that met the guideline. Additionally, as these data were collected in 2005–2006 the questions were relevant to the common technology at the time and may not have considered smart phone usage and other devices. Second, accelerometers cannot quantify water activities, movement performed when sitting (e.g.: biking), or upper body movement; cannot distinguish between quiet standing and sitting; and do not properly assess activity at very low and very high intensities. In addition, accelerometers were not given to NHANES participants in a wheelchair, which should be considered in frail populations with mobility limitations. Third, high amounts of LPA were determined using an arbitrary value based on quartiles due to the lack of clear cut-points. While these limitations may have affected the results of our study, its strengths include a large sample size, statistical analyses performed using survey weights and adjusted for potential confounding variables, and the use of accelerometer data, which is more valid than commonly used PA questionnaires [27]. This study is strengthened by its uniqueness as (1) it is the first study looking at the complete 24 H-MG and its association with frailty in a large sample of young adults and older adults, and (2) it includes behaviors such as recreational screen time, sleep and strength training that have not been extensively considered in other studies of movement behavior and frailty.

## Conclusions

In conclusion, adherence to the individual and combined components of the 24 H-MG is associated with lower frailty in adults (20–64 years) and older adults (65+). Increasing MVPA and reducing recreational screen time appears to have the greatest factors associated with frailty and should be emphasized for frailty management. Overall, there was not a clear relationship between mortality and following the complete 24 H-MG when adjusting for frailty. Future studies should investigate the longitudinal relationship between adherence to the 24 H-MG and frailty to test for a causal relationship exists between 24 H-MG adherence and frailty.

## Data Availability

The data used and/or analyzed during the current study are available from the corresponding author upon reasonable request.

## Appendix

**Table 1:** 24 h Movement guidelines and Criteria

24 H Movement Guidelines	Criteria to Meet Guideline
Moderate-to-Vigorous Physical Activity	≥ 150 min MVPA accumulated per week
Light Physical Activity	Several hours of LPA, including standing
Sedentary Time	≤ 8 h per day
Recreational Screen Time	≤ 3 h per day
Sleep	7 to 9 h for adults aged 20–64 7 to 8 h for adults aged 65+
Strength Training	≥ 2 sessions of resistance training per week

## Abbreviations

24 H-MG: 24 h Movement Guidelines
MVPA: Moderate-to-Vigorous Physical Activity
LPA: Light Physical Activity
PA: Physical Activity
NHANES: National Health and Nutrition Examination Survey
NDI: National Death Index
FI: Frailty Index
